# Examining early structural and functional brain alterations in postpartum depression through multimodal neuroimaging

**DOI:** 10.1038/s41598-021-92882-w

**Published:** 2021-06-30

**Authors:** Patricia Schnakenberg, Lisa Hahn, Susanne Stickel, Elmar Stickeler, Ute Habel, Simon B. Eickhoff, Natalia Chechko, Juergen Dukart

**Affiliations:** 1 Department of Psychiatry, Psychotherapy and Psychosomatics, Medical Faculty, Uniklinik RWTH Aachen University, Aachen, Germany; 2 Institute of Neuroscience and Medicine, Brain and Behaviour (INM-7), Research Centre Jülich, Jülich, Germany; 3 Institute of Systems Neuroscience, Medical Faculty, Heinrich Heine University Düsseldorf, Düsseldorf, Germany; 4 Department of Gynecology and Obstetrics, Medical Faculty, Uniklinik RWTH Aachen University, Aachen, Germany; 5 Institute of Neuroscience and Medicine, JARA Institute Brain Structure Function Relationship (INM-10), Research Centre Jülich, Jülich, Germany

**Keywords:** Human behaviour, Predictive markers, Depression

## Abstract

Postpartum depression (PPD) affects approximately 1 in 10 women after childbirth. A thorough understanding of a preexisting vulnerability to PPD will likely aid the early detection and treatment of PPD. Using a within-sample association, the study examined whether the brain’s structural and functional alterations predict the onset of depression. 157 euthymic postpartum women were subjected to a multimodal MRI scan within the first 6 days of childbirth and were followed up for 12 weeks. Based on a clinical interview 12 weeks postpartum, participants were classified as mentally healthy or having either PPD or adjustment disorder (AD). Voxel-based morphometry and resting-state functional connectivity comparisons were performed between the three groups. 13.4% of women in our study developed PPD (n = 21) and 12.1% (n = 19) adjustment disorder (AD). The risk factors for PPD were a psychiatric history and the experience and severity of baby blues and the history of premenstrual syndrome. Despite the different risk profiles, no differences between the PPD, AD and control group were apparent based on structural and functional neuroimaging data immediately after childbirth. At 12 weeks postpartum, a significant association was observed between Integrated Local Correlation (LCor) and the Edinburgh Postnatal Depression Score (EPDS). Our findings do not support the notion that the brain’s structural and resting-state functional alterations, if present, can be used as an early biomarker of PPD or AD. However, effects may become apparent if continuous measures of symptom severity are chosen.

## Introduction

Postpartum depression (PPD) is a disorder with the onset occurring within the first four weeks postpartum^1^. The onset of depression within the first four weeks postpartum is typically rapid, affecting particularly those with an increased sensitivity to reproductive hormone fluctuation^2,3^. Other factors such as alterations in the production of corticotropin-releasing hormone^4^ and accelerated immune responses^5^ are also thought to play a role. A history of PPD has been found to increase the mother’s risk of further depressive episodes^6^ and be associated with the child’s behavioral and emotional problems^7^. The depression-related effects on parenting^8^ significantly heighten the risk of a mother-to-child transmission of the susceptibility to depression^9,10^. An early detection of PPD, coupled with appropriate treatment measures, can not only help prevent a relapse of the condition, but also minimize the attendant emotional and financial burdens^11^.

Despite PPD being a major public health concern, about 50% of the cases go undetected, thus failing to receive evidence-based forms of treatment^12^. The earliest stages of PPD are frequently overlooked due to the commonplace nature of baby blues (sudden feelings of sadness within the first few days postpartum), affecting up to 80% of new mothers^13,14^. Another likely event linked to childbirth is adjustment disorder (AD), which is a maladaptive reaction to identifiable psychosocial stressors^1^. While baby blues only last for a brief period of time and cease within the first few days of childbirth, AD can occur up to 3 months after the exposure to psychosocial stressors^1^. The symptom severity of AD does not meet the criteria for depression at any time point, distinguishing the condition from PPD^1^. While neither AD nor baby blues have the debilitating effects of clinical depression, both should be regarded as important differential diagnoses of PPD.

Studies in major depression (MDD) have identified functional as well as structural abnormalities in the hippocampus, the amygdala, as well as the subgenual cingulate cortex (for a meta-analysis, see^15^). In contrast to studies related to MDD, imaging studies with regard to PPD are rare and frequently underpowered (for detailed reviews, see^16,17^), with only one imaging study to date including more than 14 patients^18^ and none pertaining to the structural changes in PPD^17^. In addition, the majority of the resting-state studies included PPD patients within 8 to 12 weeks postpartum (e.g.^18–22^), thereby missing any early alterations with potential prognostic value and likely mixing up early- and late-onset cases, which are thought to represent different etiologies^2^. The 4-week postpartum time frame is deemed to distinguish the so-called early-onset^2^ or hormone-sensitive phenotype of PPD^3^ from the later-onset phenotype, in which stress-inducing psychosocial factors are thought to play a more central role^2^. According to the DSM-5 criteria^1^, only an early onset can be considered as real PPD, whereas the later onset should be classified as MDD.

In spite of these limitations, functional abnormalities in PPD have been reported in the amygdala, the insula, and the orbitofrontal and dorsomedial prefrontal cortices (for a review, see^16^). However, with respect to both MDD and PPD, it is still unclear if the reported structural and functional alterations are present early in the disease course, potentially preceding the clinical symptoms, or if they develop as a consequence of the disease. An understanding of these time courses is essential to establish the diagnostic and prognostic value of the respective neuroimaging alterations.

Our study sought to detect the structural and functional brain alterations in PPD (based on the DSM-5 criteria) on the basis of multimodal neuroimaging data obtained shortly after childbirth (i.e. 1 to 6 days after delivery). The project was part of a longitudinal study aiming at early recognition of PPD (RiPoD, Risk of Postpartum Depression) in a large cohort of women who were not depressed at childbirth.

## Methods

### Study participants

The data of 157 postpartum women were used in the present study. The inclusion criteria were no depression (according to the clinical interview) at the time of recruitment, being between 18 and 45 years of age, being in the early postpartum period (1 to 6 days following childbirth), and being eligible for magnetic resonance imaging (MRI). The exclusion criteria were severe birth- and pregnancy-related complications (e.g. HELLP, eclampsia), alcoholic or psychotropic substance dependency or use during pregnancy, history of psychosis or manic episodes, and lack of sufficient command of German or English. None of the participants met the DSM-5 criteria for depression at the time of recruitment. The exclusion criteria based on the child’s condition were very premature birth (less than 29 weeks of gestation), very low birth weight (less than 1000 g), genetic defects (e.g. trisomy), or a pathological assessment on the basis of the German Child Health Test (U2).

### Procedure

Upon recruitment at the Department of Gynecology and Obstetrics, University Hospital Aachen, written informed consent was obtained from all participants. A 12-week monitoring period (T0 to T4) commenced with a clinical-anamnestic interview (T0) based on the DSM-5 criteria^1^ to obtain anamnestic and pregnancy-related information, as well as information on the current and previous psychiatric diagnoses. Additionally, a functional MRI (fMRI) assessment was conducted at T0. At time points T1 to T3, participants received links to the online platform SurveyMonkey, where they were required to fill in several questionnaires (for more detailed information on the study procedure, see supplemental information). At T4, participants were re-invited to the University Hospital Aachen for a final clinical interview (including some questionnaires) when a diagnosis of PPD or AD was made according to the DSM-5 criteria^1^. Additionally, if depressive symptomatology was present during the observational period, the Hamilton Depression Rating Scale 21 (HDR-S-21)^23^ was administered. Those who were not diagnosed with PPD or AD are referred to here as healthy controls (HC).

The study was approved by the local Ethics Committee of the University Hospital RWTH Aachen and was conducted according to the declaration of Helsinki.

### MRI procedure

The MRI scanning was conducted using a 3 Tesla Prisma MR Scanner (Siemens Medical Systems, Erlangen, Germany) located in the Medical Faculty of RWTH Aachen University. Functional images were acquired for an 11-min resting-state sequence with an echo-planar imaging (EPI) T2*-weighted contrast sequence sensitive to blood oxygen level-dependent (BOLD) contrast (34 slices, TR = 2.2 s, TE = 28 ms, FoV = 192 × 192 mm^2^, flip angle = 90°, voxel resolution = 3.0 × 3.0 × 3.0 mm^3^). T1-weighted structural images were acquired by means of a three-dimensional magnetization-prepared rapid acquisition gradient echo imaging (MPRAGE) sequence (4.12 min; 176 slices, TR = 2.3 s, TE = 1.99 ms, TI = 900 ms, FoV = 256 × 256 mm^2^, flip angle = 9°, voxel resolution = 1 × 1 × 1 mm^3^).

### Behavioral analyses

Group comparisons regarding demographic and clinical variables were conducted using chi-squared (χ^2^) tests for categorical variables and univariate analyses of variance (ANOVAs) for continuous variables. The analysis was conducted using IBM Statistics 25 (SPSS, Chicago, IL). Results are considered significant if p < 0.05.

### Resting-state preprocessing and analyses

Resting-state fMRI (rsfMRI) were preprocessed using SPM12 toolbox^24^ implemented in Matlab 2020a (MathWorks, Inc., Natick, MA). Images were realigned, unwarped, and co-registered to the structural image, spatially normalized using structural information, and smoothed by a Gaussian convolution kernel with 6 mm full-width at half maximum (FWHM). A gray matter (GM) mask was applied to reduce all analyses to GM tissue. Images were further processed in the CONN toolbox version 18.b^25^. First principal components for white matter (WM) and cerebrospinal fluid (CSF) signals as well as 24 motion parameters (Friston-24) were regressed out before computing voxel- and region-based measures of interest. Global Correlation (GCor) was calculated as the average of bivariate correlations between the BOLD signal of a given voxel and every other voxel^25^. Integrated Local Correlation (LCor) was computed as the average bivariate correlation between each voxel and its neighboring voxels weighted by a Gaussian convolution with 6 mm FWHM^26^. Fractional Amplitude of Low Frequency Fluctuations (fALFF) was calculated at each voxel as the root mean square of the BOLD signal amplitude in the analysis frequency band (here 0.01 – 0.08 Hz) divided by the amplitude in the entire frequency band^27^.

#### Voxel-based analyses

Voxel-wise group comparisons were performed in SPM12 using a flexible-factorial design for each modality with group as a factor and age as a covariate. Pair-wise t-contrasts were evaluated comparing PPD, AD and HC. All contrasts were evaluated for significance using an exact permutation-based cluster threshold (1000 permutations permuting group labels) (p < 0.05) combined with an uncorrected voxel-threshold of p < 0.01. In addition, we explored if any of the contrasts survived whole-brain voxel-wise family-wise error (FWE) correction (p < 0.05).

#### Regions of Interest (ROI) analyses

ROI analyses were performed in the CONN toolbox using the 100 regions Schaefer atlas^28^ in combination with 16 subcortical regions (right and left nucleus accumbens, amygdala, caudate, hippocampus, pallidum, putamen, thalamus, and ventral diencephalon) from the Neuromorphometrics atlas (http://neuromorphometrics.com). All pairwise group t-contrasts (i.e. HC > PPD, PPD > HC, HC > AD, AD > HC, AD > PPD, PPD > AD) were calculated for all regions in addition to the network-based statistics based on intensity.

A threshold of p < 0.01 was applied at an uncorrected level for ROI-to-ROI connections combined with a permutation-based family-wise error (FWE)-corrected cluster threshold of p < 0.05 applied at network level.

In addition, we explored if the rsfMRI data at baseline correlated with EPDS at T4 as a continuous measurement of depressive symptomatology (in contrast to a binary assignment based on the diagnosis). Therefore, a multiple regression analysis was conducted using EPDS score at T4 and age as covariates. A whole-brain voxel-wise FWE correction (p < 0.05) was applied.

### Structural data preprocessing and analysis

The structural data were preprocessed using the Computational Anatomy Toolbox (CAT12) implemented in Matlab 2020a (MathWorks, Inc., Natick, MA). The default settings of CAT12 were applied for spatial registration, segmentation and normalization with modulation. Normalized gray matter tissue volumes were smoothed with an 8 mm FWHM Gaussian kernel. After preprocessing, data were analyzed using the SPM12 toolbox implemented in Matlab 2020a (MathWorks, Inc., Natick, MA). To compare GM volumes between the groups (HC, AD, PPD), a univariate ANOVA was conducted controlling for age and total intracranial volume (TIV). T-Contrasts were used for pair-wise group comparisons. An exact permutation-based cluster threshold (p < 0.05) was applied combined with an uncorrected threshold of p < 0.01. In addition, we explored if any of the contrasts survived whole-brain voxel-wise family-wise error (FWE) correction (p < 0.05).

Additionally, we explored if the structural data at baseline correlated with EPDS at T4 as a continuous measurement of depressive symptomatology (in contrast to a binary assignment based on the diagnosis). Therefore, a multiple regression analysis was conducted using EPDS score at T4 and age as well as TIV as covariates. A whole-brain voxel-wise FWE correction (p < 0.05) was applied**.**

## Results

### Behavioral analyses

#### Demographic and clinical characteristics

Demographic, anamnestic and clinical characteristics for PPD, AD and HC are reported in Table 1. The prevalence of PPD in our study was 13.4% and the prevalence of AD was 12.1%. The EPDS score at T0 was significantly lower in HC compared to women with AD or PPD (p < 0.001). At T4, HC again showed significantly lower EPDS scores compared to the AD and the PPD groups (p < 0.001). Also, there was a significant interaction between EPDS score and group with women in the PPD group showing an increase in EPDS scores from T0 to T4, while women in the AD and HC groups showed a decrease in EPDS scores from T0 to T4, F(2,151) = 40.86, p < 0.001. Women with PPD and AD reported more often to have had a psychiatric history compared to HC (p < 0.001), while women with PPD had a psychiatric history more often than their counterparts with AD (p < 0.001). PMS severity was significantly higher in women with PPD compared to HC (p = 0.031). These women also experienced baby blues more often compared to HC (p < 0.001) and the baby blues they experienced were more severe in comparison to those experienced by HC (p = 0.001). This pattern was also apparent in the AD group: compared to HC, they experienced baby blues more often (p < 0.001) and in a more severe form (p = 0.001). Birth-related psychological or physical trauma were reported significantly more often by the AD women (36.8%) compared to PPD (14.2%) and HC (8.8%) (p = 0.002). Also, the children of women with AD were relocated to another ward significantly more often (52.6%) than those of women with PPD (19.0%) and healthy mothers (25.9%) (p = 0.034).

**Table 1:** Demographic and anamnestic data for all groups.

Variable	HC (N = 117)	PPD (N = 21)	AD (N = 19)	Statistical test
Age (M, SD)	31.97 (4.82)	31.24 (6.12)	30.68 (5.06)	F (2, 31.30) = .609, p > .05
Length of pregnancy in days (M, SD)	273.32 (14.18)	273.52 (13.81)	265.63 (23.19)	F (2, 30.78) = .982, p > .05
Birthweight of child (in gram)	3302.72 (563.13)	3320.95 (545.07)	2956.47 (911.98)	F (2, 30.85) = 1.31, p > .05
**Birth mode (yes/no)**				
Spontaneous	79/117	8/21	13/19	χ^2^ (2) = 6.87*,* p = .032*^1,3^
Ventouse	4/117	4/21	0/19	χ^2^ (2) = 10.15*,* p = .006*^1,3^
Planned C-Section	20/117	5/21	2/19	χ^2^ (2) = 1.24, p > .05
Emergency C-section	14/117	4/21	4/19	χ^2^ (2) = 1.63*,* p > .05
Married (yes/no)	82/35	14/7	13/6	χ^2^ (2) = .11*,* p > .05
Psychiatric history (yes/no)	20/96	12/9	6/13	χ^2^ (2) = 15.98*,* p < .001 *^1,2,3^
Depression	17/20	10/12	4/6	χ^2^ (2) = 12.15*,* p = .002 *^1,2,3^
Other	3/20	2/12	2/6	χ^2^ (2) = 3.84*,* p > .05
Stressful life events (yes/no)	58/59	13/8	13/6	χ^2^ (2) = 3.02*,* p > .05
Number of stressful life events (M, SD)	0.93 (1.32)	1.67 (2.13)	1.74 (1.79)	F (2, 26.56) = 2.68, p > .05
Baby blues (yes/no)	40/76	16/5	14/5	χ^2^ (2) = 19.75*,* p < .001 *^1,2^
Severity of baby blues (N = 60)	7.84 (3.80)	13.14 (2.61)	13.22 (5.04)	F (2, 13.46) = 12.14, p = .001 *^1,2^
PMS (yes/no)	47/54	12/5	11/6	χ^2^ (2) = 4.66*,* p > .05
Severity of PMS (M, SD)	7.07 (7.49)	12.47 (6.82)	7.94 (6.31)	F (2, 126) = 3.57, p = .031 *^1^
EPDS score T0 (M, SD)	4.50 (3.10)	8.19 (4.01)	9.42 (5.42)	F (2, 29.01) = 14.12, p < .001*^1,2^
EPDS score T4 (M, SD)	2.55 (2.17)	13.55 (4.50)	5.68 (4.46)	F (2, 26.56) = 59.30, p < .001*^1,2,3^
HDR-S-21 score T4 (M, SD)	–	13.74 (3.74)	–	–
Breastfeeding T0 (yes/no)	102/15	17/4	18/1	χ^2^ (2) = 1.71*,* p > .05
Birth-related psychological or physical trauma (yes/no)	9/103	3/18	7/12	χ^2^ (2) = 12.40*,* p = .002 *^1,2,3^
Relocation of child to another ward (yes/no)	30/86	4/17	10/9	χ^2^ (2) = 6.78*,* p = .034 *^2,3^

*HC* healthy controls, *PPD* postpartum depression, *AD* adjustment disorder, *M* Mean, *SD* Standard deviation, *PMS* premenstrual syndrome, *EPDS* Edinburgh Postnatal Depression Scale, *HDR-S-21* Hamilton Depression Rating Scale 21.

*Games-Howell/Bonferroni-corrected significant difference between ^1^HC and PPD, between ^2^HC and AD, and/or between ^3^AD and PPD.

### Resting-state analyses

No significant between-group differences were identified in pair-wise group comparisons for any of the voxel-wise or region-based rsfMRI measures. Applying the FWE-corrected voxel-wise threshold of p < 0.05 did not yield significant between-group differences either.

**Figure 1:**
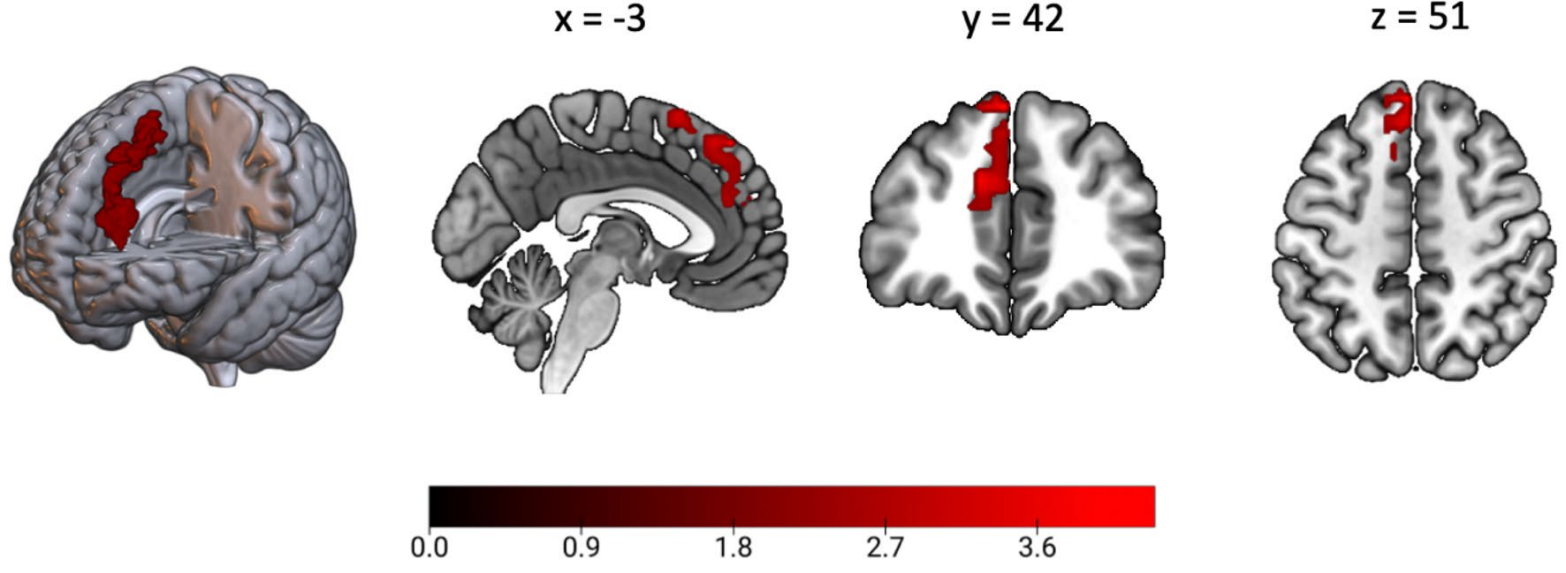
Significant positive correlation with EPDS score at T4 in the left superior medial frontal gyrus. For visualization purposes the region showing a significant whole-brain corrected voxel-wise association with EPDS at T4 is displayed at p < .01 uncorrected at voxel-level.

In additional correlational analyses, we explored if the rsfMRI measures correlated with continuous EPDS scores at T4. A whole-brain FWE-corrected significant positive correlation with EPDS score at T4 was observed for LCor in the left superior medial frontal gyrus (T(1.0, 144.0) = 5.13, MNI(x,y,z) = −3, 42, 51) (Fig. 1). No other significant associations were observed.

### Structural data analyses

No significant between-group differences were identified in pair-wise group comparisons for any of the voxel-wise or region-based rsfMRI measures using the cluster-correction threshold combined with a liberal voxel-wise threshold. Similarly, no contrast showed significant between-group differences when applying the FWE-corrected voxel-wise threshold of p < 0.05. In additional correlational analyses, we sought to determine if the structural measures correlated with continuous EPDS scores at T4 and observed no significant associations.

## Discussion

This study sought to explore early alterations in brain structure and function in PPD. The participants were recruited within a very narrow time frame following childbirth and before any clinical manifestation of PPD. The 13.4% PPD prevalence in our sample was well within the range (8% to 26%) indicated in the literature^29^. Studies that use only self-assessment tools to measure depression usually have a higher prevalence of PPD^30^ as they likely also include AD cases. In the present study, a clinical interview helped separate the cases of AD (which had a 12.1% prevalence) from those of PPD. The risk factors associated with PPD were found to be a psychiatric history, the experience and severity of baby blues and severity of PMS^31–33^. Earlier studies have shown that previous depressive episodes lead to higher depressive symptomatology shortly after childbirth (as measured with the EPDS)^34,35^. Also, a prior depressive episode and higher EPDS scores after childbirth have been found to be associated with a diagnosis of PPD^35^. In addition to the experience of PMS and baby blues being more severe in our sample of women with PPD, baby blues were also reported more often by these women, indicating a sensitivity to estrogen-mediated epigenetic changes in the early onset of PPD (for detailed reviews, see^2,17^). In contrast, women with AD reported considerably more often to have found childbirth a traumatic experience and their children were more often relocated to another ward, highlighting the reactive nature of AD symptoms. Given the transitory nature of AD, women with AD showed a decrease in their EPDS scores toward the end of the observational period. In contrast to these clear differences between the groups in terms of risk profiles and clinical scales, neither the structural nor the resting-state data could differentiate between PPD, AD, and HC. This might have been due to the fact that our study participants were not depressed when the multimodal imaging data were acquired. Multimodal neuroimaging approaches are a promising method for translating self-reported symptoms or symptoms assessed by means of clinical interviews into a neurobiological model. Applying machine-learning techniques to VBM, rsfMRI, and task-based fMRI data, a recent study has, for the first time, deciphered distinct brain signatures of schizophrenia and depression^36^. This novel approach has provided compelling evidence that the combination of neuroimaging and clinical data carries high discriminatory value in disentangling differential diagnoses. However, similar to previous studies that reported alterations in brain connectivity in PPD and assessed clinically depressed women after several weeks or months postpartum^21,22^, this study also has used data from acute psychiatric disorders^36^. Additionally, the sample sizes in these studies were small and the groups were heterogeneous in terms of onset time (e.g.^21,22,36,37^), limiting the generalizability of the findings.

The female brain undergoes dynamic neuroplastic processes during pregnancy and the postpartum period (for a review, see^38^) with decreases in GM volume in a number of brain regions (e.g. hippocampus, cingulate cortex, medial orbitofrontal cortex, insula)^39^, which have been shown to play key roles in social processes^40^, emotion regulation^41^, stress processing^42^, as well as being linked to the development of depression^15^. While these changes are thought to be adaptive, preparing new mothers for their new role, their possible contribution to the development of mental disorders cannot be ruled out^43^ as the changes in brain structure and the development of postpartum psychiatric disorders co-occur in time. However, while these adaptive processes in a postpartum brain were detectable in the structural data^39^, there was no indication of them (at least as suggested by our data) being more pronounced in women who were going to develop PPD. Research in MDD suggests dynamic changes in brain structure and function based on the state of depression (remission vs. manifest symptoms), treatment response or the severity of symptoms. For instance, in MDD, the effects of psychotherapy or pharmacotherapy are thought to be reflected in the activity patterns of the dorsal lateral prefrontal cortex and the precuneus (for a review, see^44^). Additionally, mean GM volume increases have been reported in remitted patients^45^ with the increases being particularly pronounced in the subgenual prefrontal cortex and the amygdala^46,47^. These and other studies^48,49^ indicate that alterations in brain structure and function may regress once the remission of MDD is achieved. According to these findings and our results, the observed alterations reflect a state biomarker of depression that co-occurs with the development of symptomatology. However, it is difficult to draw definite conclusions in this regard as research of trait markers or preexisting vulnerabilities with respect to both MDD and PPD is scarce. Our results indicate that trait markers, if existent, are subtle in comparison to state characteristics and may only be clearly identifiable in large groups. In our study, the only significant association between LCor and EPDS scores was observed at T4, suggesting that the effects of depressive conditions may become apparent when more sensitive continuous symptom severity measures are chosen.

In summary, the present findings support previous studies with regard to the prevalence and risk factors of PPD. Despite the disparate risk profiles of the groups in our study, no differences between the PPD, AD and control group were apparent based on the structural and functional neuroimaging data. The results indicate that if early structural or functional alterations in PPD or AD exist, they are either too subtle to be detected with the sample sizes used in our study or develop later in the disease course. More optimized longitudinal designs following larger cohorts of women from the beginning of pregnancy through childbirth into the late postpartum period may help address these questions more directly.

## Supplementary Information

Supplementary Information.

## References

[1] American Psychiatric Association. *Diagnostic and Statistical Manual of Mental Disorders (5th ed.)*. *American Journal of Psychiatry* (American Psychiatric Association, 2013). 10.1176/appi.books.9780890425596.744053.

[2] Galea LAM, Frokjaer VG (2019). Perinatal depression: Embracing variability toward better treatment and outcomes. Neuron.

[3] Bloch M, Daly RC, Rubinow DR (2003). Endocrine factors in the etiology of postpartum depression. Compr Psychiatry.

[4] Thomson M (2013). The physiological roles of placental corticotropin releasing hormone in pregnancy and childbirth. J. Physiol. Biochem..

[5] Roomruangwong C (2018). A neuro-immune, neuro-oxidative and neuro-nitrosative model of prenatal and postpartum depression. Prog. Neuro-Psychopharmacol. Biol. Psychiatry.

[6] Bloch M, Rotenberg N, Koren D, Klein E (2006). Risk factors for early postpartum depressive symptoms. Gen. Hosp. Psychiatry.

[7] Letourneau NL (2012). Postpartum depression is a family affair: addressing the impact on mothers, fathers, and children. Issues Ment. Health Nurs..

[8] Yang J (2017). Effects of parental emotional warmth on the relationship between regional gray matter volume and depression-related personality traits. Soc. Neurosci..

[9] Weissman MM (2006). Offspring of depressed parents: 20 years later. Am. J. Psychiatry.

[10] Pearson RM (2013). Maternal depression during pregnancy and the postnatal period: Risks and possible mechanisms for offspring depression at age 18 years. JAMA Psychiatry.

[11] Halfin A (2007). Depression: The benefits of early and appropriate treatment. Am. J. Manag. Care.

[12] Bauer, A., Parsonage, M., Knapp, M., Iemmi, V. & Adelaja, B. *The Costs of Perinatal Mental Health Problems* (Centre for Mental Health, 2014).

[13] O’hara MW, McCabe JE (2013). Postpartum depression: Current status and future directions. Annu. Rev. Clin. Psychol..

[14] Rezaie-Keikhaie K (2020). Systematic review and meta-analysis of the prevalence of the maternity blues in the postpartum period. J. Obstet. Gynecol. Neonatal Nurs..

[15] Gray JP, Müller VI, Eickhoff SB, Fox PT (2020). Multimodal abnormalities of brain structure and function in major depressive disorder: A meta-analysis of neuroimaging studies. Am. J. Psychiatry.

[16] Stickel S (2019). Neural correlates of depression in women across the reproductive lifespan—An fMRI review. J. Affect. Disord..

[17] Sacher J, Chechko N, Dannlowski U, Walter M, Derntl B (2020). The peripartum human brain: current understanding and future perspectives. Front. Neuroendocrinol..

[18] Deligiannidis KM (2019). Resting-state functional connectivity, cortical GABA, and neuroactive steroids in peripartum and peripartum depressed women: A functional magnetic resonance imaging and spectroscopy study. Neuropsychopharmacology.

[19] Wonch KE (2016). Postpartum depression and brain response to infants: Differential amygdala response and connectivity. Soc. Neurosci..

[20] Epperson CN (2006). Preliminary evidence of reduced occipital GABA concentrations in puerperal women: A 1H-MRS study. Psychopharmacol. Berl..

[21] Deligiannidis KM (2013). GABAergic neuroactive steroids and resting-state functional connectivity in postpartum depression: A preliminary study. J. Psychiatr. Res..

[22] Chase HW, Moses-Kolko EL, Zevallos C, Wisner KL, Phillips ML (2014). Disrupted posterior cingulate-amygdala connectivity in postpartum depressed women as measured with resting BOLD fMRI. Soc. Cogn. Affect. Neurosci..

[23] Hamilton M (1960). The Hamilton Depression Scale—Accelerator or break on antidepressant drug discovery. Psychiatry.

[24] Penny, W. D., Friston, K. J., Ashburner, J. T., Kiebel, S. J. & Nichols, T. E. *Statistical Parametric Mapping: The Analysis of Functional Brain Images*. (Elsevier, 2011).

[25] Whitfield-Gabrieli S, Nieto-Castanon A (2012). Conn: A functional connectivity toolbox for correlated and anticorrelated brain networks. Brain Connect..

[26] Deshpande G, LaConte S, Peltier S, Hu X (2009). Integrated local correlation: A new measure of local coherence in fMRI data. Hum. Brain Mapp..

[27] Zou Q-H (2008). An improved approach to detection of amplitude of low-frequency fluctuation (ALFF) for resting-state fMRI: Fractional ALFF. J. Neurosci. Methods.

[28] Schaefer A (2018). Local-global parcellation of the human cerebral cortex from intrinsic functional connectivity MRI. Cereb. cortex.

[29] Shorey S (2018). Prevalence and incidence of postpartum depression among healthy mothers: A systematic review and meta-analysis. J. Psychiatr. Res..

[30] Fisher J (2012). Prevalence and determinants of common perinatal mental disorders in women in low-and lower-middle-income countries: A systematic review. Bull. World Health Organ..

[31] Buttner MM (2013). Examination of premenstrual symptoms as a risk factor for depression in postpartum women. Arch. Womens. Ment. Health.

[32] Robertson E, Grace S, Wallington T, Stewart DE (2004). Antenatal risk factors for postpartum depression: a synthesis of recent literature. Gen. Hosp. Psychiatry.

[33] Henshaw C, Foreman D, Cox J (2004). Postnatal blues: A risk factor for postnatal depression. J. Psychosom. Obstet. Gynecol..

[34] Schnakenberg P (2021). The early postpartum period–Differences between women with and without a history of depression. J. Psychiatr. Res..

[35] El-Hachem C (2014). Early identification of women at risk of postpartum depression using the Edinburgh Postnatal Depression Scale (EPDS) in a sample of Lebanese women. BMC Psychiatry.

[36] Stoyanov D (2020). Multivariate analysis of structural and functional neuroimaging can inform psychiatric differential diagnosis. Diagnostics.

[37] Xiao-juan W, Jian W, Zhi-hong L, Yan M, Shi-wei Z (2011). Increased posterior cingulate, medial frontal and decreased temporal regional homogeneity in depressed mothers. A resting-state functional magnetic resonance study. Procedia Environ. Sci..

[38] Leuner B, Sabihi S (2016). The birth of new neurons in the maternal brain: Hormonal regulation and functional implications. Front. Neuroendocrinol..

[39] Hoekzema E (2017). Pregnancy leads to long-lasting changes in human brain structure. Nat. Neurosci..

[40] Alcalá-López D (2018). Computing the social brain connectome across systems and states. Cereb. cortex.

[41] Morawetz C, Bode S, Derntl B, Heekeren HR (2017). The effect of strategies, goals and stimulus material on the neural mechanisms of emotion regulation: A meta-analysis of fMRI studies. Neurosci. Biobehav. Rev..

[42] Chattarji S, Tomar A, Suvrathan A, Ghosh S, Rahman MM (2015). Neighborhood matters: Divergent patterns of stress-induced plasticity across the brain. Nat. Neurosci..

[43] Barba-Müller E, Craddock S, Carmona S, Hoekzema E (2019). Brain plasticity in pregnancy and the postpartum period: Links to maternal caregiving and mental health. Arch. Womens. Ment. Health.

[44] Graham J (2013). Meta-analytic evidence for neuroimaging models of depression: State or trait?. J. Affect. Disord..

[45] Phillips JL, Batten LA, Aldosary F, Tremblay P, Blier P (2012). Brain-volume increase with sustained remission in patients with treatment-resistant unipolar depression. J. Clin. Psychiatry.

[46] Yucel K (2009). Increased subgenual prefrontal cortex size in remitted patients with major depressive disorder. Psychiatry Res. Neuroimaging.

[47] Yüksel D (2018). Longitudinal brain volume changes in major depressive disorder. J. Neural Transm..

[48] Dohm K, Redlich R, Zwitserlood P, Dannlowski U (2017). Trajectories of major depression disorders: A systematic review of longitudinal neuroimaging findings. Aust. New Zeal. J. Psychiatry.

[49] Li CT (2010). Structural and cognitive deficits in remitting and non-remitting recurrent depression: A voxel-based morphometric study. Neuroimage.

