# Supplementary material for: Examining early structural and functional brain alterations in postpartum depression through multimodal neuroimaging
Source: Sci Rep. 2021 Jun 30;11:13551. doi: 10.1038/s41598-021-92882-w (PMC8245412; doi:10.1038/s41598-021-92882-w)
Supplement: Supplementary file 1 — Supplementary Information. [file 41598_2021_92882_MOESM1_ESM.docx]

**Supplemental information**


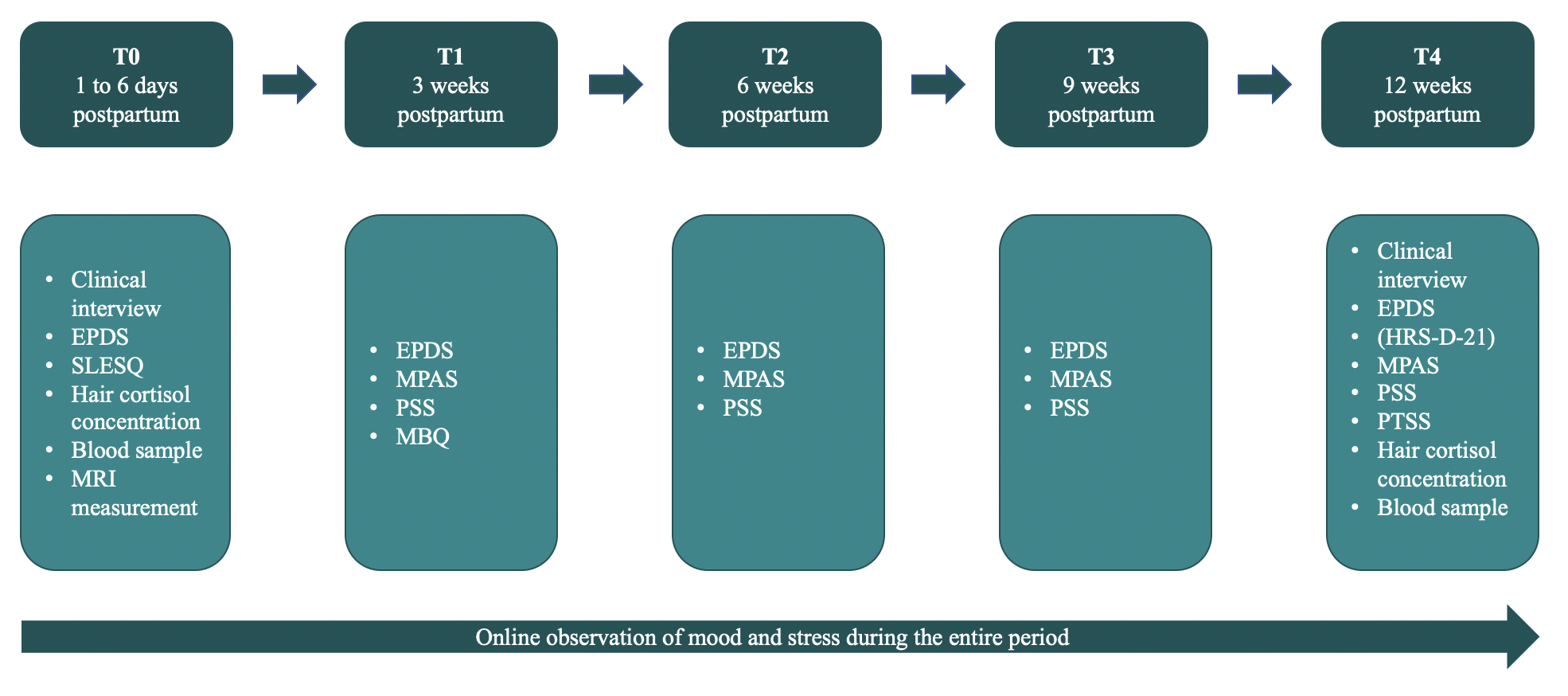
The questionnaires used in the study included the Edinburgh Postnatal Depression Scale (EPDS; timepoint T0-T4) ^1^ and the Stressful Life Events Screening Questionnaire (SLESQ; timepoint T0) ^2^. Additionally, the Maternity Blues questionnaire (MBQ; timepoint T1) was used to assess the presence and severity of baby blues ^3^ and the Premenstrual Tension Syndrome Scale (PTSS; timepoint T4) ^4^ to examine the symptoms of premenstrual syndrome. At timepoints T1 to T4, mother-child attachment was assessed using the Maternal Postpartum Attachment Scale (MPAS) ^5^ and perceived stress was assessed with the Perceived Stress Scale (PSS) ^6^. On a bi-daily basis during the 12-week period, women answered questions regarding their mood and their stress level. For an overview of the study procedure, see Figure 2.

Figure 2. Overview of the study procedure

Note: EPDS = Edinburgh Postnatal Depression Scale, SLESQ = Stressful Life Events Screening Questionnaire, MPAS = Maternal Postpartum Attachment Scale, PSS = Perceived Stress Scale, MBQ = Maternity blues questionnaire, HRS-D-21= Hamilton Depression Rating Scale 21, PTSS = Premenstrual Tension Syndrome Scale

**References**

1. Cox, J. L., Holden, J. M. & Sagovsky, R. Detection of postnatal depression: development of the 10-item Edinburgh Postnatal Depression Scale. *Br. J. psychiatry* **150**, 782–786 (1987).

2. Goodman, L. A., Corcoran, C., Turner, K., Yuan, N. & Green, B. L. Assessing traumatic event exposure: General issues and preliminary findings for the Stressful Life Events Screening Questionnaire. *J. Trauma. Stress Off. Publ. Int. Soc. Trauma. Stress Stud.* **11**, 521–542 (1998).

3. Kennerley, H. & Gath, D. Maternity blues: I. Detection and measurement by questionnaire. *Br. J. Psychiatry* **155**, 356–362 (1989).

4. Steiner, M., Haskett, R. F. & Carroll, B. J. Premenstrual tension syndrome: the development of research diagnostic criteria and new rating scales. *Acta Psychiatr. Scand.* **62**, 177–190 (1980).

5. Condon, J. T. & Corkindale, C. J. The assessment of parent-to-infant attachment: Development of a self-report questionnaire instrument. *J. Reprod. Infant Psychol.* **16**, 57–76 (1998).

6. Klein, E. M. *et al.* The German version of the Perceived Stress Scale–psychometric characteristics in a representative German community sample. *BMC Psychiatry* **16**, 159 (2016).
